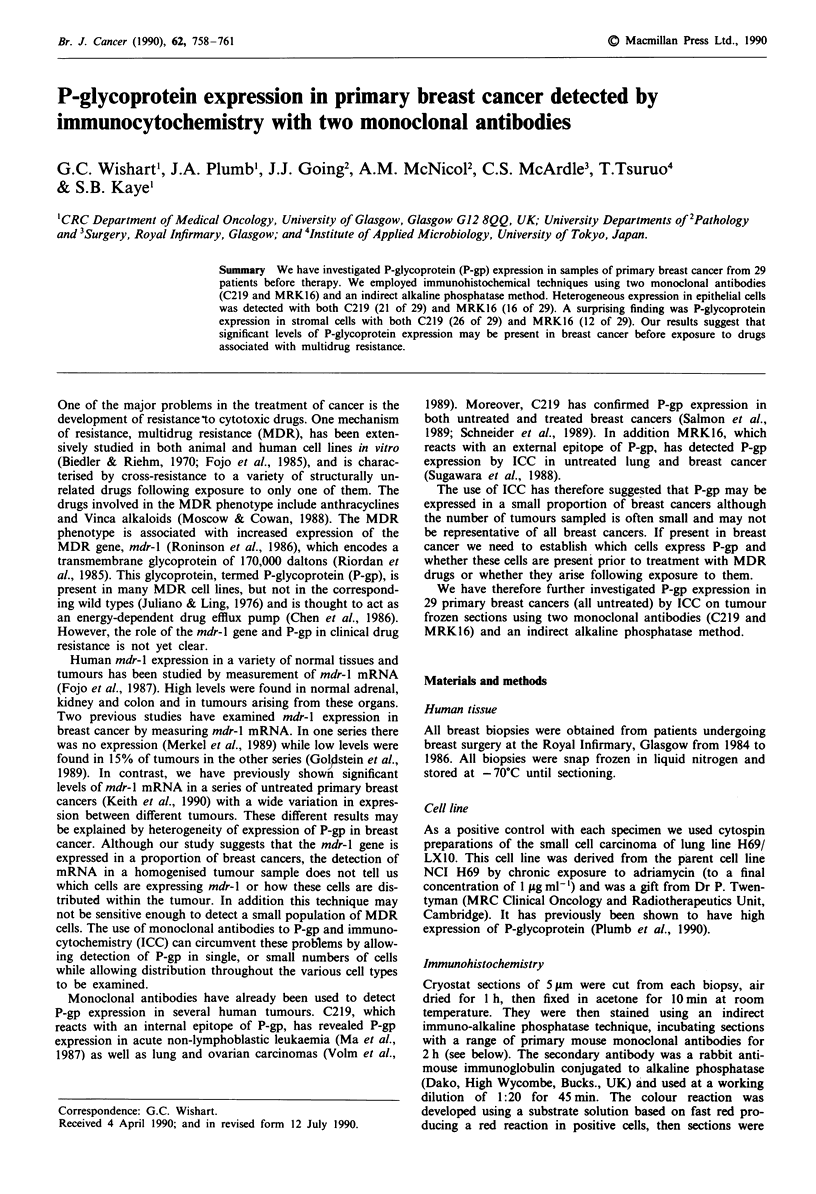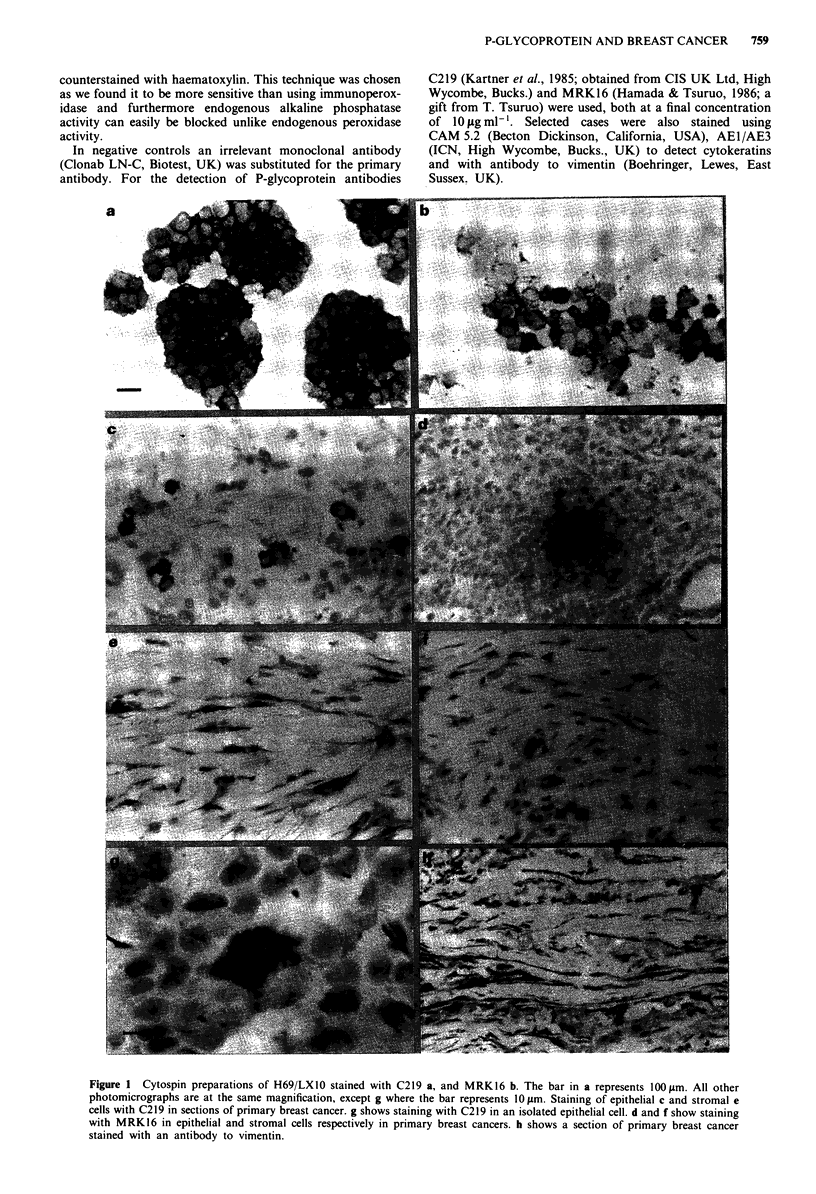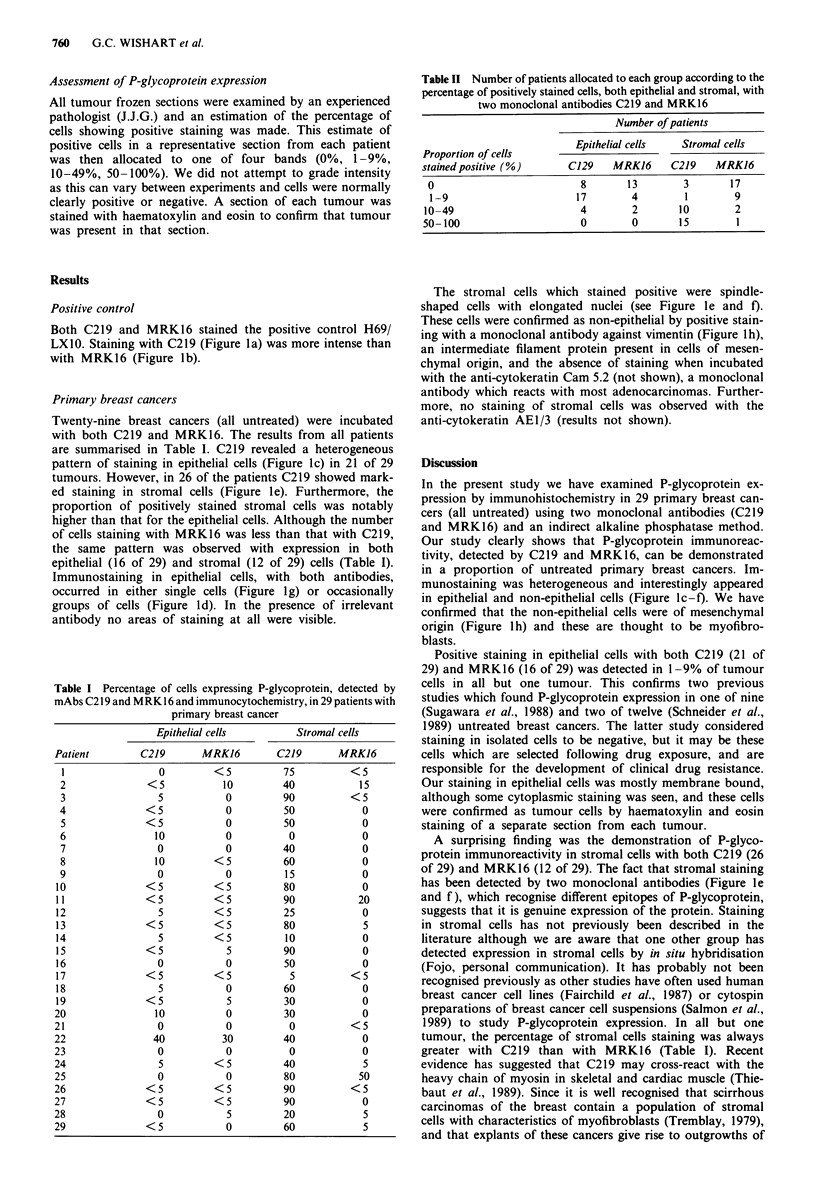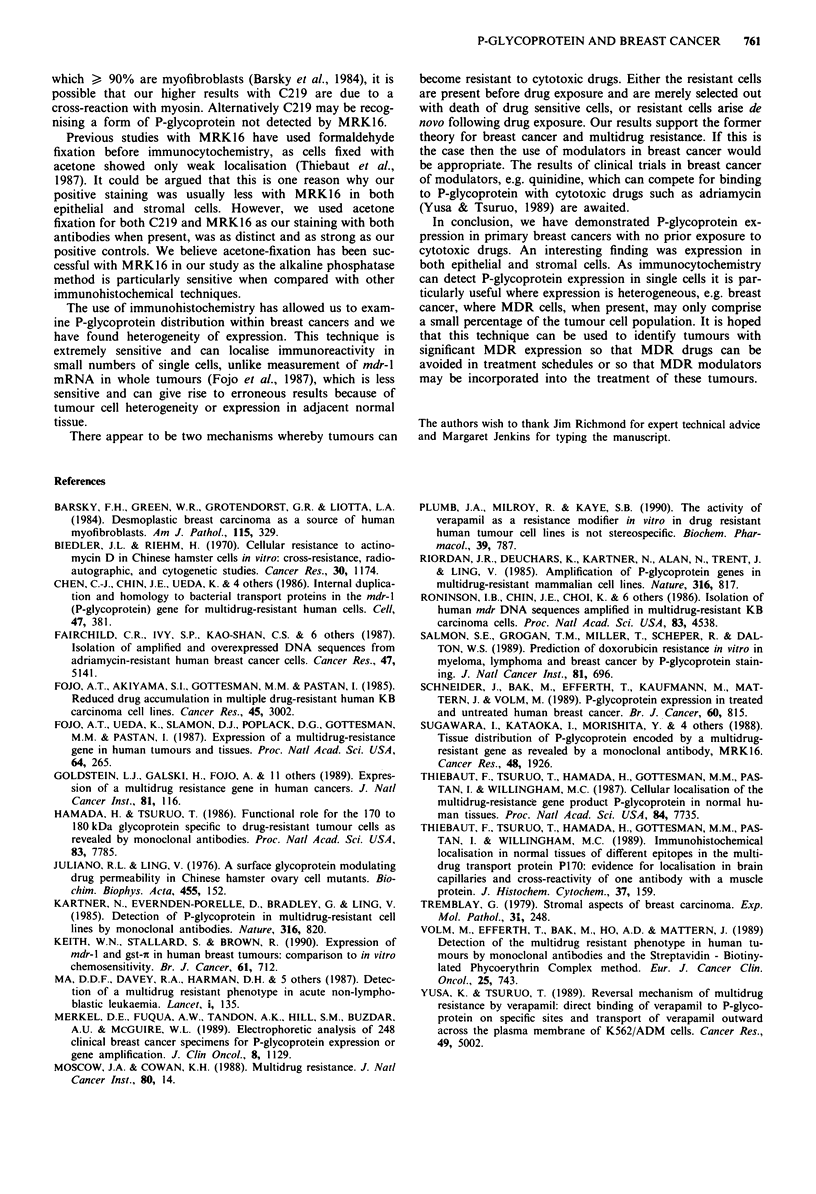# P-glycoprotein expression in primary breast cancer detected by immunocytochemistry with two monoclonal antibodies.

**DOI:** 10.1038/bjc.1990.373

**Published:** 1990-11

**Authors:** G. C. Wishart, J. A. Plumb, J. J. Going, A. M. McNicol, C. S. McArdle, T. Tsuruo, S. B. Kaye

**Affiliations:** CRC Department of Medical Oncology, University of Glasgow, UK.

## Abstract

**Images:**


Br. J. Cancer (1990), 62, 758-761
© Macmillan Press Ltd., 1990

### P-glycoprotein expression in primary breast cancer detected by immunocytochemistry with two monoclonal antibodies

G.C. Wishart[1], J.A. Plumb[1], J.J. Going[2], A.M. McNicol[2], C.S. McArdle[3], T.Tsuruo[4] & S.B. Kaye[1]

[1]*CRC Department of Medical Oncology, University of Glasgow, Glasgow G12 8QQ, UK; University Departments of* [2]*Pathology and* [3]*Surgery, Royal Infirmary, Glasgow; and* [4]*Institute of Applied Microbiology, University of Tokyo, Japan.*

**Summary** We have investigated P-glycoprotein (P-gp) expression in samples of primary breast cancer from 29 patients before therapy. We employed immunohistochemical techniques using two monoclonal antibodies (C219 and MRK16) and an indirect alkaline phosphatase method. Heterogeneous expression in epithelial cells was detected with both C219 (21 of 29) and MRK16 (16 of 29). A surprising finding was P-glycoprotein expression in stromal cells with both C219 (26 of 29) and MRK16 (12 of 29). Our results suggest that significant levels of P-glycoprotein expression may be present in breast cancer before exposure to drugs associated with multidrug resistance.

One of the major problems in the treatment of cancer is the development of resistance to cytotoxic drugs. One mechanism of resistance, multidrug resistance (MDR), has been extensively studied in both animal and human cell lines *in vitro* (Biedler & Riehm, 1970; Fojo *et al.*, 1985), and is characterised by cross-resistance to a variety of structurally unrelated drugs following exposure to only one of them. The drugs involved in the MDR phenotype include anthracyclines and Vinca alkaloids (Moscow & Cowan, 1988). The MDR phenotype is associated with increased expression of the MDR gene, *mdr*-1 (Roninson *et al.*, 1986), which encodes a transmembrane glycoprotein of 170,000 daltons (Riordan *et al.*, 1985). This glycoprotein, termed P-glycoprotein (P-gp), is present in many MDR cell lines, but not in the corresponding wild types (Juliano & Ling, 1976) and is thought to act as an energy-dependent drug efflux pump (Chen *et al.*, 1986). However, the role of the *mdr*-1 gene and P-gp in clinical drug resistance is not yet clear.

Human *mdr*-1 expression in a variety of normal tissues and tumours has been studied by measurement of *mdr*-1 mRNA (Fojo *et al.*, 1987). High levels were found in normal adrenal, kidney and colon and in tumours arising from these organs. Two previous studies have examined *mdr*-1 expression in breast cancer by measuring *mdr*-1 mRNA. In one series there was no expression (Merkel *et al.*, 1989) while low levels were found in 15% of tumours in the other series (Goldstein *et al.*, 1989). In contrast, we have previously shown significant levels of *mdr*-1 mRNA in a series of untreated primary breast cancers (Keith *et al.*, 1990) with a wide variation in expression between different tumours. These different results may be explained by heterogeneity of expression of P-gp in breast cancer. Although our study suggests that the *mdr*-1 gene is expressed in a proportion of breast cancers, the detection of mRNA in a homogenised tumour sample does not tell us which cells are expressing *mdr*-1 or how these cells are distributed within the tumour. In addition this technique may not be sensitive enough to detect a small population of MDR cells. The use of monoclonal antibodies to P-gp and immunocytochemistry (ICC) can circumvent these problems by allowing detection of P-gp in single, or small numbers of cells while allowing distribution throughout the various cell types to be examined.

Monoclonal antibodies have already been used to detect P-gp expression in several human tumours. C219, which reacts with an internal epitope of P-gp, has revealed P-gp expression in acute non-lymphoblastic leukaemia (Ma *et al.*, 1987) as well as lung and ovarian carcinomas (Volm *et al.*, 1989). Moreover, C219 has confirmed P-gp expression in both untreated and treated breast cancers (Salmon *et al.*, 1989; Schneider *et al.*, 1989). In addition MRK16, which reacts with an external epitope of P-gp, has detected P-gp expression by ICC in untreated lung and breast cancer (Sugawara *et al.*, 1988).

The use of ICC has therefore suggested that P-gp may be expressed in a small proportion of breast cancers although the number of tumours sampled is often small and may not be representative of all breast cancers. If present in breast cancer we need to establish which cells express P-gp and whether these cells are present prior to treatment with MDR drugs or whether they arise following exposure to them.

We have therefore further investigated P-gp expression in 29 primary breast cancers (all untreated) by ICC on tumour frozen sections using two monoclonal antibodies (C219 and MRK16) and an indirect alkaline phosphatase method.

### Materials and methods

### *Human tissue*

All breast biopsies were obtained from patients undergoing breast surgery at the Royal Infirmary, Glasgow from 1984 to 1986. All biopsies were snap frozen in liquid nitrogen and stored at $-70°C$ until sectioning.

### *Cell line*

As a positive control with each specimen we used cytospin preparations of the small cell carcinoma of lung line H69/LX10. This cell line was derived from the parent cell line NCI H69 by chronic exposure to adriamycin (to a final concentration of $1\ \mu g\ ml^{-1}$) and was a gift from Dr P. Twentyman (MRC Clinical Oncology and Radiotherapeutics Unit, Cambridge). It has previously been shown to have high expression of P-glycoprotein (Plumb *et al.*, 1990).

### *Immunohistochemistry*

Cryostat sections of $5\ \mu m$ were cut from each biopsy, air dried for 1 h, then fixed in acetone for 10 min at room temperature. They were then stained using an indirect immuno-alkaline phosphatase technique, incubating sections with a range of primary mouse monoclonal antibodies for 2 h (see below). The secondary antibody was a rabbit anti-mouse immunoglobulin conjugated to alkaline phosphatase (Dako, High Wycombe, Bucks., UK) and used at a working dilution of 1:20 for 45 min. The colour reaction was developed using a substrate solution based on fast red producing a red reaction in positive cells, then sections were

Correspondence: G.C. Wishart.
Received 4 April 1990; and in revised form 12 July 1990.

counterstained with haematoxylin. This technique was chosen as we found it to be more sensitive than using immunoperoxidase and furthermore endogenous alkaline phosphatase activity can easily be blocked unlike endogenous peroxidase activity.

In negative controls an irrelevant monoclonal antibody (Clonab LN-C, Biotest, UK) was substituted for the primary antibody. For the detection of P-glycoprotein antibodies C219 (Kartner *et al.*, 1985; obtained from CIS UK Ltd, High Wycombe, Bucks.) and MRK16 (Hamada & Tsuruo, 1986; a gift from T. Tsuruo) were used, both at a final concentration of $10\,\mu g\,ml^{-1}$. Selected cases were also stained using CAM 5.2 (Becton Dickinson, California, USA), AE1/AE3 (ICN, High Wycombe, Bucks., UK) to detect cytokeratins and with antibody to vimentin (Boehringer, Lewes, East Sussex, UK).

**Figure 1** Cytospin preparations of H69/LX10 stained with C219 **a**, and MRK16 **b**. The bar in **a** represents 100 μm. All other photomicrographs are at the same magnification, except **g** where the bar represents 10 μm. Staining of epithelial **c** and stromal **e** cells with C219 in sections of primary breast cancer. **g** shows staining with C219 in an isolated epithelial cell. **d** and **f** show staining with MRK16 in epithelial and stromal cells respectively in primary breast cancers. **h** shows a section of primary breast cancer stained with an antibody to vimentin.

*Assessment of P-glycoprotein expression*

All tumour frozen sections were examined by an experienced pathologist (J.J.G.) and an estimation of the percentage of cells showing positive staining was made. This estimate of positive cells in a representative section from each patient was then allocated to one of four bands (0%, 1–9%, 10–49%, 50–100%). We did not attempt to grade intensity as this can vary between experiments and cells were normally clearly positive or negative. A section of each tumour was stained with haematoxylin and eosin to confirm that tumour was present in that section.

### Results

*Positive control*

Both C219 and MRK16 stained the positive control H69/LX10. Staining with C219 (Figure 1a) was more intense than with MRK16 (Figure 1b).

*Primary breast cancers*

Twenty-nine breast cancers (all untreated) were incubated with both C219 and MRK16. The results from all patients are summarised in Table I. C219 revealed a heterogeneous pattern of staining in epithelial cells (Figure 1c) in 21 of 29 tumours. However, in 26 of the patients C219 showed marked staining in stromal cells (Figure 1e). Furthermore, the proportion of positively stained stromal cells was notably higher than that for the epithelial cells. Although the number of cells staining with MRK16 was less than that with C219, the same pattern was observed with expression in both epithelial (16 of 29) and stromal (12 of 29) cells (Table I). Immunostaining in epithelial cells, with both antibodies, occurred in either single cells (Figure 1g) or occasionally groups of cells (Figure 1d). In the presence of irrelevant antibody no areas of staining at all were visible.

**Table I** Percentage of cells expressing P-glycoprotein, detected by mAbs C219 and MRK16 and immunocytochemistry, in 29 patients with primary breast cancer

| | *Epithelial cells* | | *Stromal cells* | |
|---|---|---|---|---|
| *Patient* | *C219* | *MRK16* | *C219* | *MRK16* |
| 1 | 0 | <5 | 75 | <5 |
| 2 | <5 | 10 | 40 | 15 |
| 3 | 5 | 0 | 90 | <5 |
| 4 | <5 | 0 | 50 | 0 |
| 5 | <5 | 0 | 50 | 0 |
| 6 | 10 | 0 | 0 | 0 |
| 7 | 0 | 0 | 40 | 0 |
| 8 | 10 | <5 | 60 | 0 |
| 9 | 0 | 0 | 15 | 0 |
| 10 | <5 | <5 | 80 | 0 |
| 11 | <5 | <5 | 90 | 20 |
| 12 | 5 | <5 | 25 | 0 |
| 13 | <5 | <5 | 80 | 5 |
| 14 | 5 | <5 | 10 | 0 |
| 15 | <5 | 5 | 90 | 0 |
| 16 | 0 | 0 | 50 | 0 |
| 17 | <5 | <5 | 5 | <5 |
| 18 | 5 | 0 | 60 | 0 |
| 19 | <5 | 5 | 30 | 0 |
| 20 | 10 | 0 | 30 | 0 |
| 21 | 0 | 0 | 0 | <5 |
| 22 | 40 | 30 | 40 | 0 |
| 23 | 0 | 0 | 0 | 0 |
| 24 | 5 | <5 | 40 | 5 |
| 25 | 0 | 0 | 80 | 50 |
| 26 | <5 | <5 | 90 | <5 |
| 27 | <5 | <5 | 90 | 0 |
| 28 | 0 | 5 | 20 | 5 |
| 29 | <5 | 0 | 60 | 5 |

**Table II** Number of patients allocated to each group according to the percentage of positively stained cells, both epithelial and stromal, with two monoclonal antibodies C219 and MRK16

| | *Number of patients* | | | |
|---|---|---|---|---|
| | *Epithelial cells* | | *Stromal cells* | |
| *Proportion of cells stained positive (%)* | *C129* | *MRK16* | *C219* | *MRK16* |
| 0 | 8 | 13 | 3 | 17 |
| 1–9 | 17 | 4 | 1 | 9 |
| 10–49 | 4 | 2 | 10 | 2 |
| 50–100 | 0 | 0 | 15 | 1 |

The stromal cells which stained positive were spindle-shaped cells with elongated nuclei (see Figure 1e and f). These cells were confirmed as non-epithelial by positive staining with a monoclonal antibody against vimentin (Figure 1h), an intermediate filament protein present in cells of mesenchymal origin, and the absence of staining when incubated with the anti-cytokeratin Cam 5.2 (not shown), a monoclonal antibody which reacts with most adenocarcinomas. Furthermore, no staining of stromal cells was observed with the anti-cytokeratin AE1/3 (results not shown).

### Discussion

In the present study we have examined P-glycoprotein expression by immunohistochemistry in 29 primary breast cancers (all untreated) using two monoclonal antibodies (C219 and MRK16) and an indirect alkaline phosphatase method. Our study clearly shows that P-glycoprotein immunoreactivity, detected by C219 and MRK16, can be demonstrated in a proportion of untreated primary breast cancers. Immunostaining was heterogeneous and interestingly appeared in epithelial and non-epithelial cells (Figure 1c–f). We have confirmed that the non-epithelial cells were of mesenchymal origin (Figure 1h) and these are thought to be myofibroblasts.

Positive staining in epithelial cells with both C219 (21 of 29) and MRK16 (16 of 29) was detected in 1–9% of tumour cells in all but one tumour. This confirms two previous studies which found P-glycoprotein expression in one of nine (Sugawara *et al.*, 1988) and two of twelve (Schneider *et al.*, 1989) untreated breast cancers. The latter study considered staining in isolated cells to be negative, but it may be these cells which are selected following drug exposure, and are responsible for the development of clinical drug resistance. Our staining in epithelial cells was mostly membrane bound, although some cytoplasmic staining was seen, and these cells were confirmed as tumour cells by haematoxylin and eosin staining of a separate section from each tumour.

A surprising finding was the demonstration of P-glycoprotein immunoreactivity in stromal cells with both C219 (26 of 29) and MRK16 (12 of 29). The fact that stromal staining has been detected by two monoclonal antibodies (Figure 1e and f), which recognise different epitopes of P-glycoprotein, suggests that it is genuine expression of the protein. Staining in stromal cells has not previously been described in the literature although we are aware that one other group has detected expression in stromal cells by *in situ* hybridisation (Fojo, personal communication). It has probably not been recognised previously as other studies have often used human breast cancer cell lines (Fairchild *et al.*, 1987) or cytospin preparations of breast cancer cell suspensions (Salmon *et al.*, 1989) to study P-glycoprotein expression. In all but one tumour, the percentage of stromal cells staining was always greater with C219 than with MRK16 (Table I). Recent evidence has suggested that C219 may cross-react with the heavy chain of myosin in skeletal and cardiac muscle (Thiebaut *et al.*, 1989). Since it is well recognised that scirrhous carcinomas of the breast contain a population of stromal cells with characteristics of myofibroblasts (Tremblay, 1979), and that explants of these cancers give rise to outgrowths of which ≥ 90% are myofibroblasts (Barsky *et al.*, 1984), it is possible that our higher results with C219 are due to a cross-reaction with myosin. Alternatively C219 may be recognising a form of P-glycoprotein not detected by MRK16.

Previous studies with MRK16 have used formaldehyde fixation before immunocytochemistry, as cells fixed with acetone showed only weak localisation (Thiebaut *et al.*, 1987). It could be argued that this is one reason why our positive staining was usually less with MRK16 in both epithelial and stromal cells. However, we used acetone fixation for both C219 and MRK16 as our staining with both antibodies when present, was as distinct and as strong as our positive controls. We believe acetone-fixation has been successful with MRK16 in our study as the alkaline phosphatase method is particularly sensitive when compared with other immunohistochemical techniques.

The use of immunohistochemistry has allowed us to examine P-glycoprotein distribution within breast cancers and we have found heterogeneity of expression. This technique is extremely sensitive and can localise immunoreactivity in small numbers of single cells, unlike measurement of *mdr*-1 mRNA in whole tumours (Fojo *et al.*, 1987), which is less sensitive and can give rise to erroneous results because of tumour cell heterogeneity or expression in adjacent normal tissue.

There appear to be two mechanisms whereby tumours can become resistant to cytotoxic drugs. Either the resistant cells are present before drug exposure and are merely selected out with death of drug sensitive cells, or resistant cells arise *de novo* following drug exposure. Our results support the former theory for breast cancer and multidrug resistance. If this is the case then the use of modulators in breast cancer would be appropriate. The results of clinical trials in breast cancer of modulators, e.g. quinidine, which can compete for binding to P-glycoprotein with cytotoxic drugs such as adriamycin (Yusa & Tsuruo, 1989) are awaited.

In conclusion, we have demonstrated P-glycoprotein expression in primary breast cancers with no prior exposure to cytotoxic drugs. An interesting finding was expression in both epithelial and stromal cells. As immunocytochemistry can detect P-glycoprotein expression in single cells it is particularly useful where expression is heterogeneous, e.g. breast cancer, where MDR cells, when present, may only comprise a small percentage of the tumour cell population. It is hoped that this technique can be used to identify tumours with significant MDR expression so that MDR drugs can be avoided in treatment schedules or so that MDR modulators may be incorporated into the treatment of these tumours.

The authors wish to thank Jim Richmond for expert technical advice and Margaret Jenkins for typing the manuscript.

### References

BARSKY, F.H., GREEN, W.R., GROTENDORST, G.R. & LIOTTA, L.A. (1984). Desmoplastic breast carcinoma as a source of human myofibroblasts. *Am J. Pathol.*, **115**, 329.

BIEDLER, J.L. & RIEHM, H. (1970). Cellular resistance to actinomycin D in Chinese hamster cells *in vitro*: cross-resistance, radioautographic, and cytogenetic studies. *Cancer Res.*, **30**, 1174.

CHEN, C.-J., CHIN, J.E., UEDA, K. & 4 others (1986). Internal duplication and homology to bacterial transport proteins in the *mdr*-1 (P-glycoprotein) gene for multidrug-resistant human cells. *Cell*, **47**, 381.

FAIRCHILD, C.R., IVY, S.P., KAO-SHAN, C.S. & 6 others (1987). Isolation of amplified and overexpressed DNA sequences from adriamycin-resistant human breast cancer cells. *Cancer Res.*, **47**, 5141.

FOJO, A.T., AKIYAMA, S.I., GOTTESMAN, M.M. & PASTAN, I. (1985). Reduced drug accumulation in multiple drug-resistant human KB carcinoma cell lines. *Cancer Res.*, **45**, 3002.

FOJO, A.T., UEDA, K., SLAMON, D.J., POPLACK, D.G., GOTTESMAN, M.M. & PASTAN, I. (1987). Expression of a multidrug-resistance gene in human tumours and tissues. *Proc. Natl Acad. Sci. USA*, **64**, 265.

GOLDSTEIN, L.J., GALSKI, H., FOJO, A. & 11 others (1989). Expression of a multidrug resistance gene in human cancers. *J. Natl Cancer Inst.*, **81**, 116.

HAMADA, H. & TSURUO, T. (1986). Functional role for the 170 to 180 kDa glycoprotein specific to drug-resistant tumour cells as revealed by monoclonal antibodies. *Proc. Natl Acad. Sci. USA*, **83**, 7785.

JULIANO, R.L. & LING, V. (1976). A surface glycoprotein modulating drug permeability in Chinese hamster ovary cell mutants. *Biochim. Biophys. Acta*, **455**, 152.

KARTNER, N., EVERNDEN-PORELLE, D., BRADLEY, G. & LING, V. (1985). Detection of P-glycoprotein in multidrug-resistant cell lines by monoclonal antibodies. *Nature*, **316**, 820.

KEITH, W.N., STALLARD, S. & BROWN, R. (1990). Expression of *mdr*-1 and gst-π in human breast tumours: comparison to *in vitro* chemosensitivity. *Br. J. Cancer*, **61**, 712.

MA, D.D.F., DAVEY, R.A., HARMAN, D.H. & 5 others (1987). Detection of a multidrug resistant phenotype in acute non-lymphoblastic leukaemia. *Lancet*, **i**, 135.

MERKEL, D.E., FUQUA, A.W., TANDON, A.K., HILL, S.M., BUZDAR, A.U. & MCGUIRE, W.L. (1989). Electrophoretic analysis of 248 clinical breast cancer specimens for P-glycoprotein expression or gene amplification. *J. Clin Oncol.*, **8**, 1129.

MOSCOW, J.A. & COWAN, K.H. (1988). Multidrug resistance. *J. Natl Cancer Inst.*, **80**, 14.

PLUMB, J.A., MILROY, R. & KAYE, S.B. (1990). The activity of verapamil as a resistance modifier *in vitro* in drug resistant human tumour cell lines is not stereospecific. *Biochem. Pharmacol.*, **39**, 787.

RIORDAN, J.R., DEUCHARS, K., KARTNER, N., ALAN, N., TRENT, J. & LING, V. (1985). Amplification of P-glycoprotein genes in multidrug-resistant mammalian cell lines. *Nature*, **316**, 817.

RONINSON, I.B., CHIN, J.E., CHOI, K. & 6 others (1986). Isolation of human *mdr* DNA sequences amplified in multidrug-resistant KB carcinoma cells. *Proc. Natl Acad. Sci. USA*, **83**, 4538.

SALMON, S.E., GROGAN, T.M., MILLER, T., SCHEPER, R. & DALTON, W.S. (1989). Prediction of doxorubicin resistance *in vitro* in myeloma, lymphoma and breast cancer by P-glycoprotein staining. *J. Natl Cancer Inst.*, **81**, 696.

SCHNEIDER, J., BAK, M., EFFERTH, T., KAUFMANN, M., MATTERN, J. & VOLM, M. (1989). P-glycoprotein expression in treated and untreated human breast cancer. *Br. J. Cancer*, **60**, 815.

SUGAWARA, I., KATAOKA, I., MORISHITA, Y. & 4 others (1988). Tissue distribution of P-glycoprotein encoded by a multidrug-resistant gene as revealed by a monoclonal antibody, MRK16. *Cancer Res.*, **48**, 1926.

THIEBAUT, F., TSURUO, T., HAMADA, H., GOTTESMAN, M.M., PASTAN, I. & WILLINGHAM, M.C. (1987). Cellular localisation of the multidrug-resistance gene product P-glycoprotein in normal human tissues. *Proc. Natl Acad. Sci. USA*, **84**, 7735.

THIEBAUT, F., TSURUO, T., HAMADA, H., GOTTESMAN, M.M., PASTAN, I. & WILLINGHAM, M.C. (1989). Immunohistochemical localisation in normal tissues of different epitopes in the multidrug transport protein P170: evidence for localisation in brain capillaries and cross-reactivity of one antibody with a muscle protein. *J. Histochem. Cytochem.*, **37**, 159.

TREMBLAY, G. (1979). Stromal aspects of breast carcinoma. *Exp. Mol. Pathol.*, **31**, 248.

VOLM, M., EFFERTH, T., BAK, M., HO, A.D. & MATTERN, J. (1989) Detection of the multidrug resistant phenotype in human tumours by monoclonal antibodies and the Streptavidin - Biotinylated Phycoerythrin Complex method. *Eur. J. Cancer Clin. Oncol.*, **25**, 743.

YUSA, K. & TSURUO, T. (1989). Reversal mechanism of multidrug resistance by verapamil: direct binding of verapamil to P-glycoprotein on specific sites and transport of verapamil outward across the plasma membrane of K562/ADM cells. *Cancer Res.*, **49**, 5002.